# Assessing Diagnostic Precision and Therapeutic Guidance Using Artificial Intelligence in Functional Neurosurgery Cases

**DOI:** 10.7759/cureus.83592

**Published:** 2025-05-06

**Authors:** Filipi Fim Andreão, Matheus Moura Nascimento, André M De Faria, Filipe Virgilio Ribeiro, Otavio Augusto da Costa, Lucca B Palavani, Guilherme Santos Piedade, Alexis Morell, Timoteo Almeida, Pedro Henrique Martins da Cunha, Ricardo J Komotar, Joacir Graciolli Cordeiro, Bernardo Assumpcao de Monaco

**Affiliations:** 1 Department of Neurosurgery, Federal University of Rio de Janeiro, Rio de Janeiro, BRA; 2 Faculty of Medicine, University Center of Maceio, Maceió, BRA; 3 Medicine, Federal Hospital for State Employees, Rio de Janeiro, BRA; 4 Faculty of Medicine, Barão de Mauá Faculty of Medicine, Ribeirão Preto, BRA; 5 Faculty of Medicine, Pontifical Catholic University of Campinas, Campinas, BRA; 6 Faculty of Medicine, Max Planck University Center, Indaiatuba, BRA; 7 Department of Neurosurgery, University of Miami Leonard M. Miller School of Medicine, Miami, USA; 8 Department of Critical Care Medicine, University of Pittsburgh, Pittsburgh, USA; 9 Department of Neurosurgery, University of Miami, Miami, USA; 10 Department of Radiation Oncology, University of Miami, Miami, USA; 11 Department of Neurosurgery, University of São Paulo, São Paulo, BRA; 12 Neurological Surgery, University of Miami Leonard M. Miller School of Medicine, Miami, USA; 13 Neurological Surgery, University of Miami, Miami, USA; 14 Neurosurgery, University of São Paulo, São Paulo, BRA

**Keywords:** ai models, artificial intelligence, functional neurosurgery, machine learning, neurosurgery

## Abstract

Background and objective

The integration of artificial intelligence (AI) into functional neurosurgery holds great promise for improving diagnostic precision and therapeutic decision-making. This study aimed to assess the diagnostic accuracy and treatment recommendations provided by five AI models - ChatGPT-3.5, ChatGPT-4, Perplexity, Gemini, and AtlasGPT - when applied to complex clinical cases.

Methods

Ten clinical cases related to functional neurosurgery were selected from the medical literature to minimize ambiguity and ensure clarity. Each case was presented to the AI models with the directive to propose a diagnosis and therapeutic approach, using medical terminology. The AI responses were evaluated by a panel of seven functional neurosurgeons, who scored the accuracy of diagnoses and treatment recommendations on a scale from 0 to 10. The scores were analyzed using one-way ANOVA, with post-hoc analysis via Tukey’s test to identify significant differences among the AI models.

Results

Diagnostic accuracy varied significantly among the AI models. AtlasGPT achieved a median diagnostic score of 9 [quartile 1 (Q1): 9, quartile 3 (Q3): 10, interquartile range (IQR): 1], demonstrating superior performance compared to Perplexity, which had a median score of 9 with a higher IQR of 3 (p=0.04), and ChatGPT-3.5, which had a median score of 10 but with a lower IQR of 2 (p=0.03). In terms of treatment recommendations, AtlasGPT's median score was 8, notably higher than ChatGPT-3.5, which had a median score of 7 (p<0.01), and Perplexity, which also had a median score of 8 (p<0.01).

Conclusions

This study's findings underscore the potential of AI models in functional neurosurgery, particularly in enhancing diagnostic accuracy and expanding therapeutic options. However, the variability in performance among different AI systems suggests the need for continuous evaluation and refinement of these technologies. Rigorous assessment and interdisciplinary collaboration are essential to ensure the safe and effective integration of AI into clinical practice.

## Introduction

The term artificial intelligence (AI) was first coined during the Dartmouth Summer Workshop in 1956, where the technology was widely referred to as "thinking machines". In simple terms, AI can be defined as the capability of a machine to learn and recognize patterns and relationships from a sufficient number of representative examples, and to use this information effectively for decision-making based on unknown data [1]. Despite the extensive media attention given to AI, many healthcare professionals still perceive AI as a "black box," leading to both inflated expectations and unfounded fears [2].

With the advent of new AI technologies, the medical environment has undergone significant transformations. Patient diagnosis, based on radiological, pathological, endoscopic, ultrasonographic, and biochemical tests, has been enhanced with greater accuracy and reduced human workload. Furthermore, medical treatments during the perioperative period, which includes preoperative preparation, the surgical phase, and postoperative recovery, have witnessed significant improvements, resulting in more favorable surgical outcomes. AI technology has also played a crucial role in other areas, such as drug production, medical management, and medical education, steering these fields toward new approaches and solutions [3].

Functional neurosurgery, a complex and continuously evolving subspecialty, has been pivotal in treating neurological disorders that affect vital functions such as movement, sensory perception, and behavior. With the advent of AI in the field of medicine, new possibilities have emerged for enhancing diagnostic accuracy and therapeutic efficacy, especially in areas where clinical complexity challenges even the most experienced specialists. This study aims to explore the capabilities of various AI models - ChatGPT-3.5, ChatGPT-4, Perplexity, Gemini, and AtlasGPT - in formulating diagnoses and developing treatment plans for complex clinical cases in functional neurosurgery. The research addresses not only the accuracy of the AI models but also their ability to integrate clinical and radiological data in high-complexity contexts, a crucial competency in functional neurosurgery.

## Materials and methods

Ten clinical cases related to functional neurosurgery were selected from the medical literature [4] to ensure clarity and reduce ambiguity, making the diagnosis and treatment as straightforward as possible. Each clinical case was presented to five AI systems: ChatGPT-3.5, ChatGPT-4, Perplexity, Gemini, and AtlasGPT. Before presenting each case, the following directive was given to the AI systems: "Imagine yourself as a functional neurosurgeon and propose the diagnosis and therapeutic approach for the patient. Subsequently, share your conclusions in a precise and specific manner, using scientific and medical terminology to detail the treatment and diagnosis." This directive was followed by the presentation of the case and two questions regarding the diagnosis and the most indicated treatment. It is noteworthy to say that the prompt did not limit AI’s answers to FDA-approved treatments. Radiological images were provided to the AI systems for cases that included such images, utilizing the upload option where available.

The responses from the AI systems were collected and then evaluated by a panel of seven functional neurosurgeons. Each response was scored on a scale from 0 to 10, assessing both the diagnosis and the treatment recommendation. The collected scores were subjected to statistical analysis. The analysis involved conducting a one-way ANOVA test to identify any statistically significant differences between the AI systems' performance. Median, quartile 1 (Q1), quartile 3 (Q3), and interquartile range (IQR) were used to statistically represent the scores of each AI. Further analysis entailed a post-hoc analysis using Tukey’s test to identify specific differences by comparing each AI system against the others. A p-value <0.05 and a 95% confidence interval (CI) were used as the criteria for significance. RStudio version 2023.12.1+402 "Ocean Storm"; the R packages ggplot2, dplyr, gridExtra, and grid were utilized for data visualization and analysis.

This study did not involve the use of human subjects or patient data, and it solely utilized clinical cases that are publicly available in the medical literature. Therefore, no institutional review board (IRB) or ethics committee approval was required.

## Results

Baseline diagnosis and treatment: AI models' responses

Table 1 presents a detailed comparison of diagnoses and treatments for various neurosurgical functional disorders as determined by different AI models, including AtlasGPT, Perplexity, ChatGPT 3.5, and ChatGPT 4, alongside original clinical decisions. Overall, AI models frequently align with the original diagnoses, such as identifying Parkinson's disease in a case initially diagnosed as advanced Parkinsonism. Treatment recommendations from AI models showed some variations. For instance, while most models suggested deep brain stimulation (DBS) for Parkinson's disease, treatments for medically refractory epilepsy varied, with recommendations including responsive neurostimulation (RNS) and laser interstitial thermal therapy (LITT). The models consistently suggested treatments that aligned with or expanded upon the original treatment plans, indicating a level of agreement among AI models in their clinical decision-making capabilities.

**Table 1:** Baseline characteristics of the included cases and AI models' responses CPSP: central post-stroke pain; DBS: deep brain stimulation; DREZ: dorsal root entry zone; LITT: laser interstitial thermal therapy; MR: magnetic resonance; MVD: microvascular decompression; RNS: responsive neurostimulation; SRS: stereotactic radiosurgery; STN: subthalamic nucleus; TN: trigeminal neuralgia

Case	Original diagnosis	Original treatment	AtlasGPT diagnosis	AtlasGPT treatment	Perplexity diagnosis	Perplexity treatment	ChatGPT-3.5 diagnosis	ChatGPT-3.5 treatment	ChatGPT-4 diagnosis	ChatGPT-4 treatment
1	Advanced parkinsonism	Bilateral STN DBS	Parkinson's disease	STN DBS	Parkinson's disease	DBS	Parkinson's disease	DBS	Parkinson's disease	DBS
2	Medically refractory epilepsy	Vagal nerve stimulator	Refractory epilepsy	RNS	Medically refractory epilepsy	LITT	Medically refractory epilepsy	LITT or RNS	Drug-resistant epilepsy	Minimally invasive surgery: LITT or SRS might be considered if a focal lesion becomes identifiable. RNS system
3	Temporal lobe epilepsy	Complete anterior temporal lobectomy	Right mesial temporal lobe epilepsy	Anteromedial temporal lobectomy	Mesial temporal lobe epilepsy	Anterior temporal lobectomy with amygdalohippocampectomy	Mesial temporal lobe epilepsy	Anterior temporal lobectomy or selective amygdalohippocampectomy	Temporal lobe epilepsy	Anterior temporal lobectomy or selective amygdalohippocampectomy
4	Essential tremor	DBS targeting the ventral intermediate STN bilaterally	Bilateral upper extremity action tremor	DBS and thalamotomy	Essential tremor with alcohol-responsive tremor subtype	DBS-focused ultrasound thalamotomy	Essential tremor	Targeting the ventral intermediate STN with DBS	Essential tremor	DBS: targeting the ventral intermediate STN
5	Type I TN	Initially, carbamazepine (Tegretol), in case of failure of medications, MVD via the retrosigmoidal approach	TN	Neurovascular compression, MVD	TN	MVD	TN secondary to vascular compression	MVD	TN	MVD
6	Intractable pain syndrome likely from deafferentation from his prior stroke	Multidisciplinary treatment prior to consideration for motor cortex stimulation	CPSP or thalamic pain syndrome	Motor cortex stimulation and DBS	CPSP, also known as thalamic pain syndrome	DBS	Post-stroke neuropathic pain	DBS	CPSP- thalamic pain syndrome	Motor cortex stimulation or DBS
7	Deafferentation pain of the right upper extremity	DREZ lesioning	Neuropathic pain refractory to conservative management	Neuromodulation: spinal cord stimulation or DREZ lesioning	Brachial plexus avulsion showing evidence of pseudomeningoceles from C5 to T1 on the right side and a flail arm	Consider surgical options such as pseudomeningocele repair, neuroma excision, or spinal cord stimulation	Neuropathic pain	Surgical Intervention: neurostimulation	Post-traumatic brachial plexus injury leading to a flail arm, associated with severe neuropathic pain	DREZ lesioning procedure to target the sensory pathways at the root entry zone
8	Medically refractory epilepsy that appears to be of extratemporal origin	Craniotomy and cortical grid placement, antiepileptic drug, and/or placement of vagal nerve stimulator should be considered	Focal epilepsy with secondary generalization	Resective surgery	Drug-resistant epilepsy	Surgical resection of the epileptogenic focus is a viable option	Refractory epilepsy	Surgical resection of the epileptogenic focus through a procedure such as a focal cortical resection or epilepsy surgery	Drug-resistant focal epilepsy	Resective surgery such as a lobectomy or lesionectomy or RNS or DBS
9	Hypothalamic hamartoma causing gelastic seizures	Open surgical resection, SRS, and MR-guided laser ablation	Hypothalamic hamartoma	MR-guided LITT or SRS	Hypothalamic hamartoma	Surgical resection; in cases where complete resection is not feasible due to the deep location of the tumor, SRS may be considered	Hypothalamic hamartoma	Microsurgical resection or disconnection of the hamartoma from surrounding structures	Hypothalamic hamartoma	Resection or disconnection procedures: endoscopic or open surgical approaches for resecting or disconnecting the hamartoma from surrounding structures, SRS
10	Classic TN	Open surgery, or needle-based procedures (radiofrequency ablation, glycerol rhizotomy, or balloon compression)	TN	Percutaneous treatments or SRS	TN	MVD	TN	MVD	TN	MVD, balloon compression, glycerol rhizotomy, or radiofrequency thermal rhizotomy, SRS

AI models' diagnosis evaluation by case

**Figure 1:**
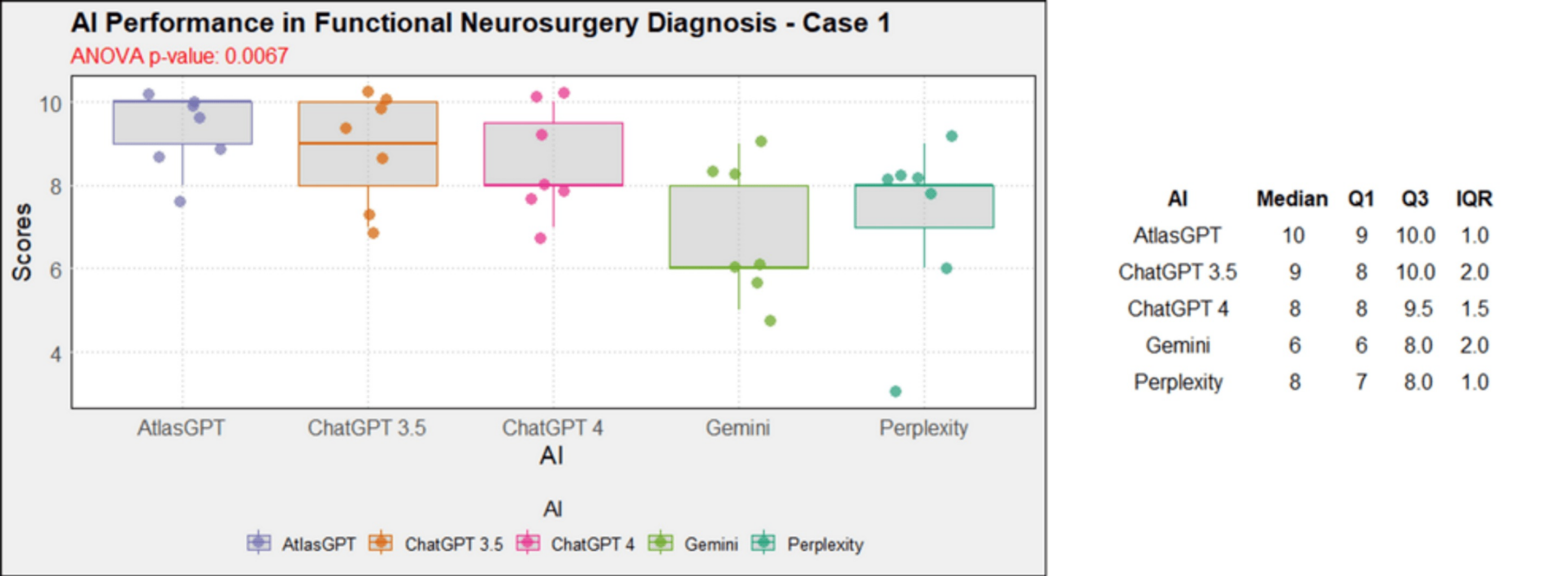
AI models' performance in functional neurosurgery diagnosis - case 1 AI: artificial intelligence; ANOVA: analysis of variance; IQR: interquartile range; Q1: quartile 1; Q3: quartile 3

**Figure 2:**
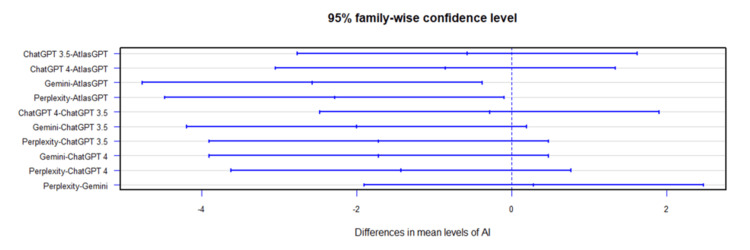
Summary of differences between the performance of AI models AI: artificial intelligence

**Figure 3:**
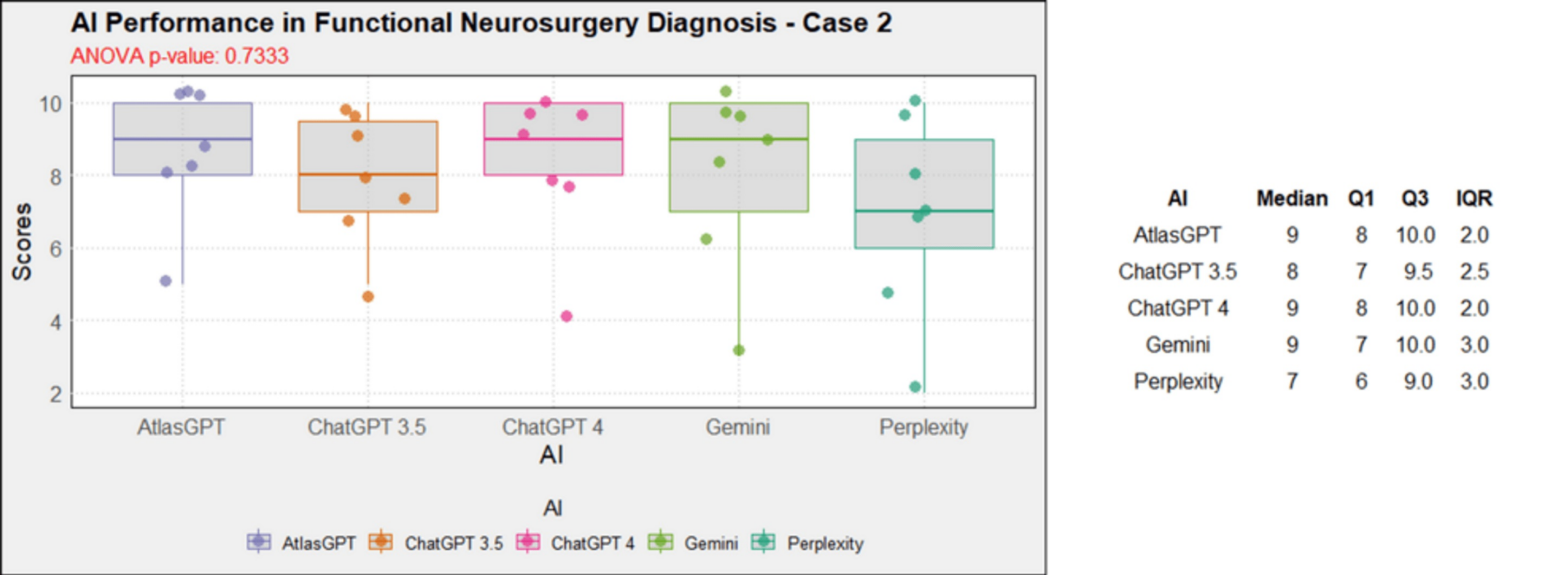
AI models' performance in functional neurosurgery diagnosis - case 2 AI: artificial intelligence; ANOVA: analysis of variance; IQR: interquartile range; Q1: quartile 1; Q3: quartile 3

**Figure 4:**
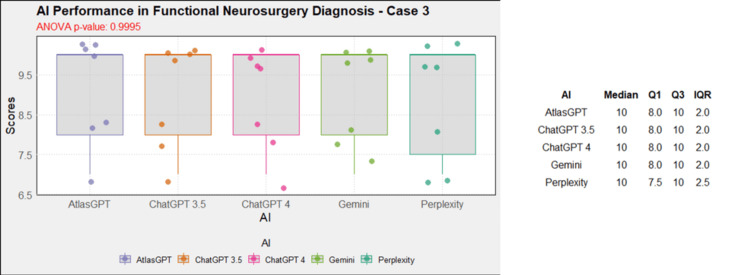
AI models' performance in functional neurosurgery diagnosis - case 3 AI: artificial intelligence; ANOVA: analysis of variance; IQR: interquartile range; Q1: quartile 1; Q3: quartile 3

**Figure 5:**
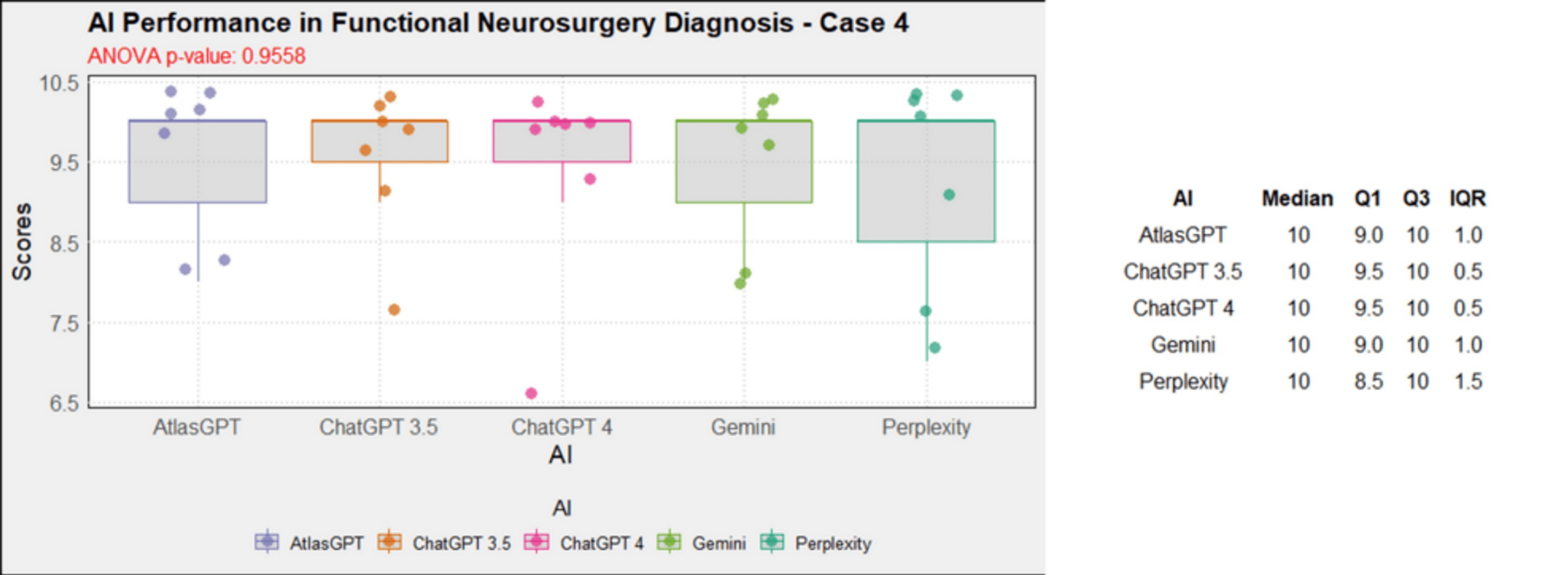
AI models' performance in functional neurosurgery diagnosis - case 4 AI: artificial intelligence; ANOVA: analysis of variance; IQR: interquartile range; Q1: quartile 1; Q3: quartile 3

**Figure 6:**
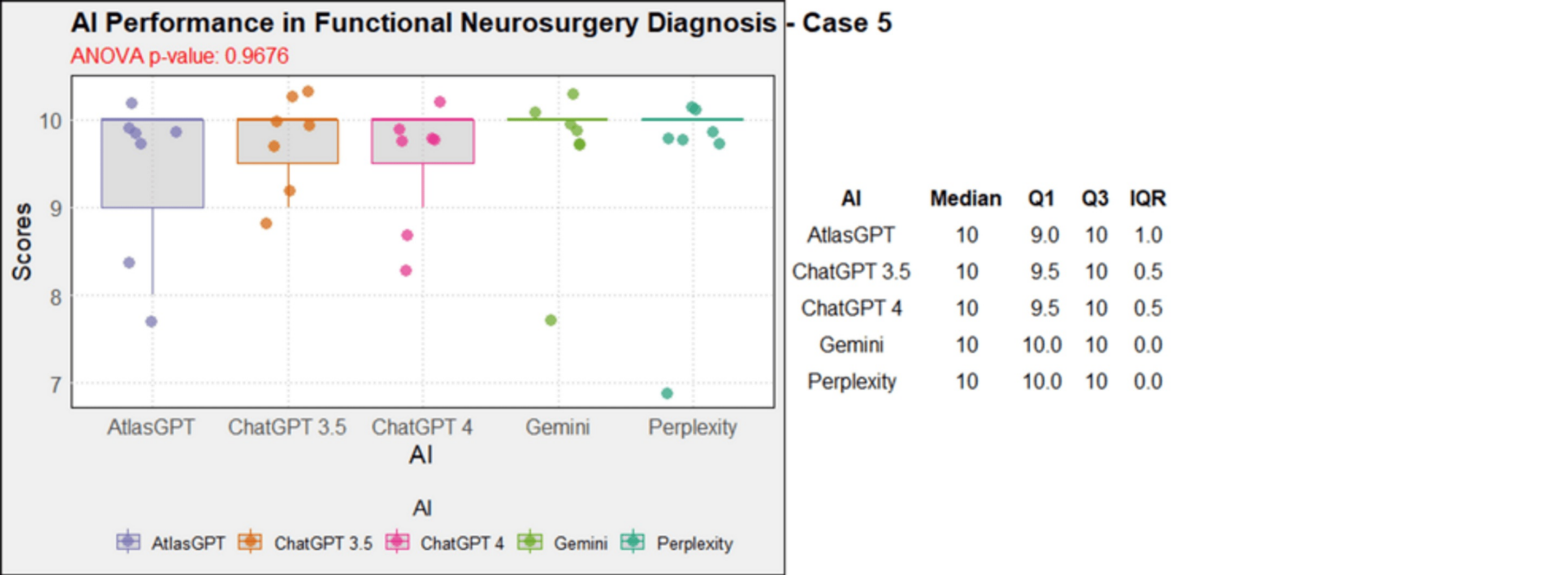
AI models' performance in functional neurosurgery diagnosis - case 5 AI: artificial intelligence; ANOVA: analysis of variance; IQR: interquartile range; Q1: quartile 1; Q3: quartile 3

**Figure 7:**
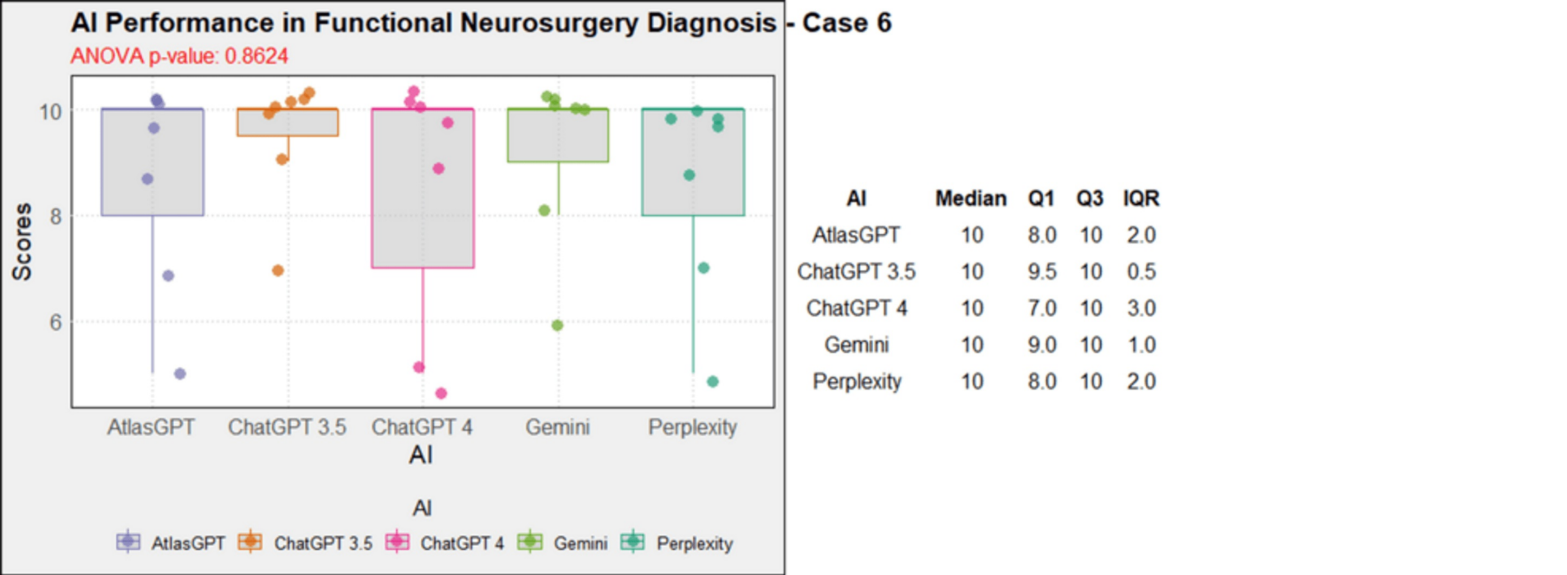
AI models' performance in functional neurosurgery diagnosis - case 6 AI: artificial intelligence; ANOVA: analysis of variance; IQR: interquartile range; Q1: quartile 1; Q3: quartile 3

**Figure 8:**
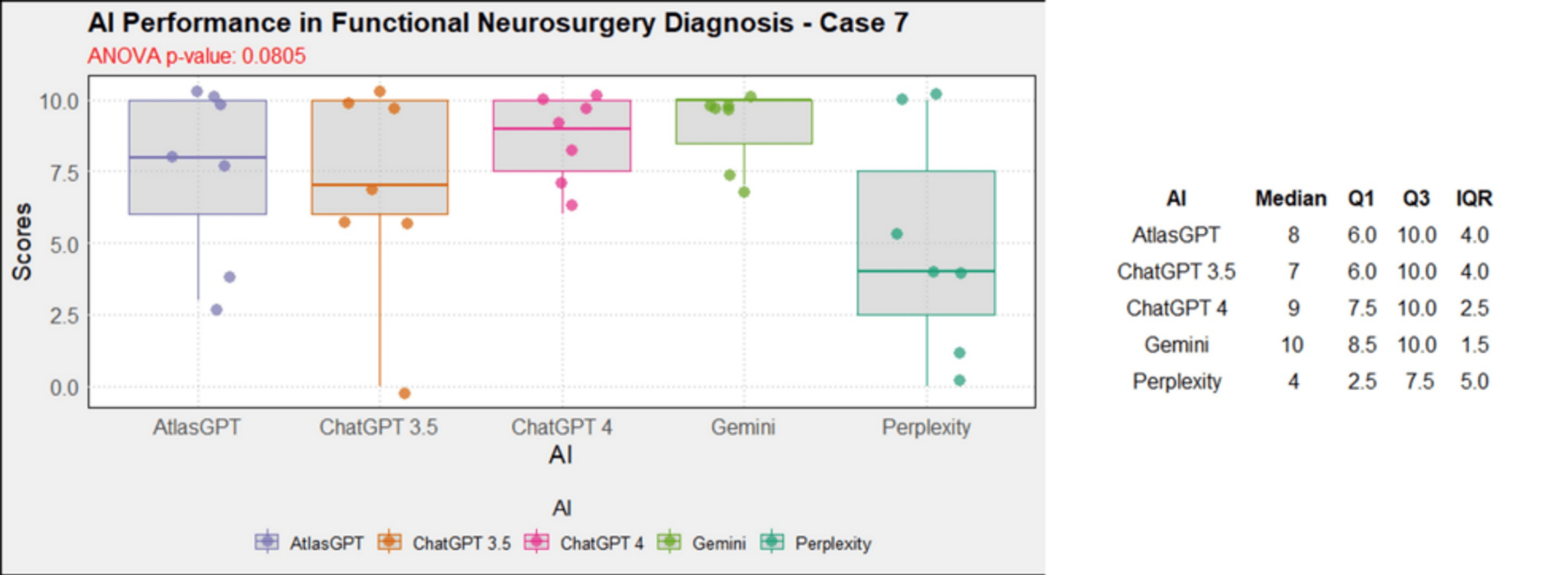
AI models' performance in functional neurosurgery diagnosis - case 7 AI: artificial intelligence; ANOVA: analysis of variance; IQR: interquartile range; Q1: quartile 1; Q3: quartile 3

**Figure 9:**
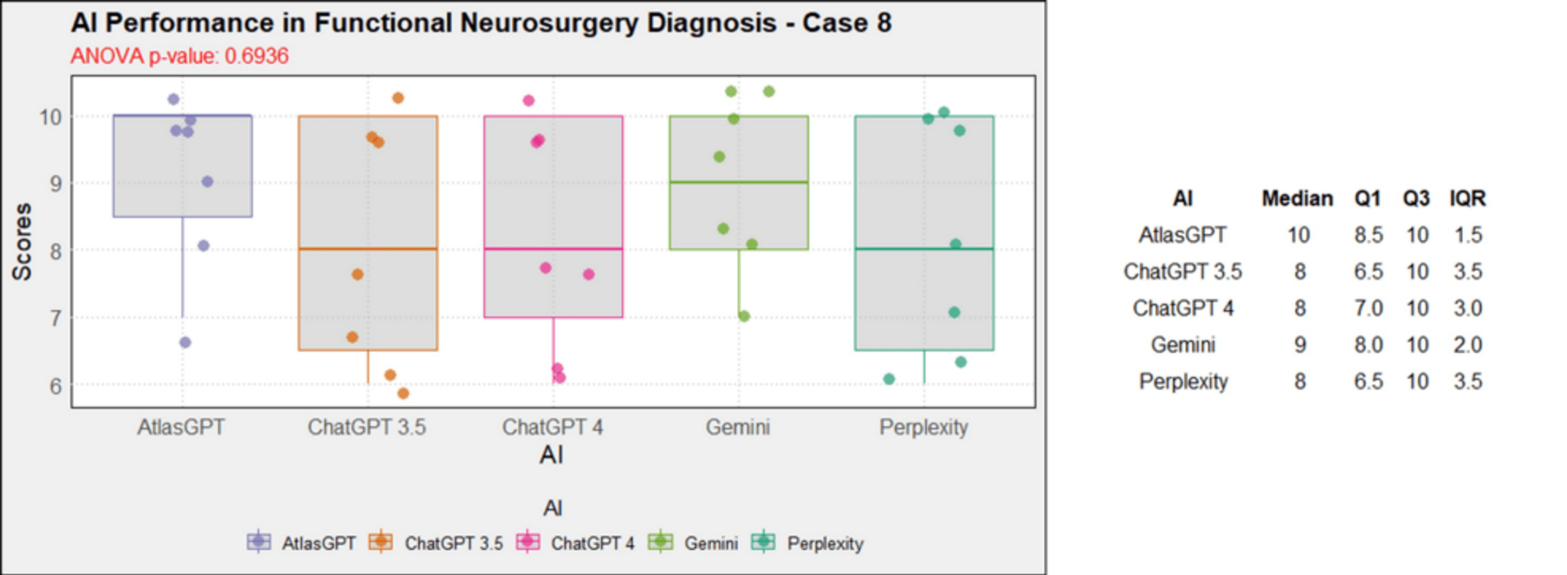
AI models' performance in functional neurosurgery diagnosis - case 8 AI: artificial intelligence; ANOVA: analysis of variance; IQR: interquartile range; Q1: quartile 1; Q3: quartile 3

**Figure 10:**
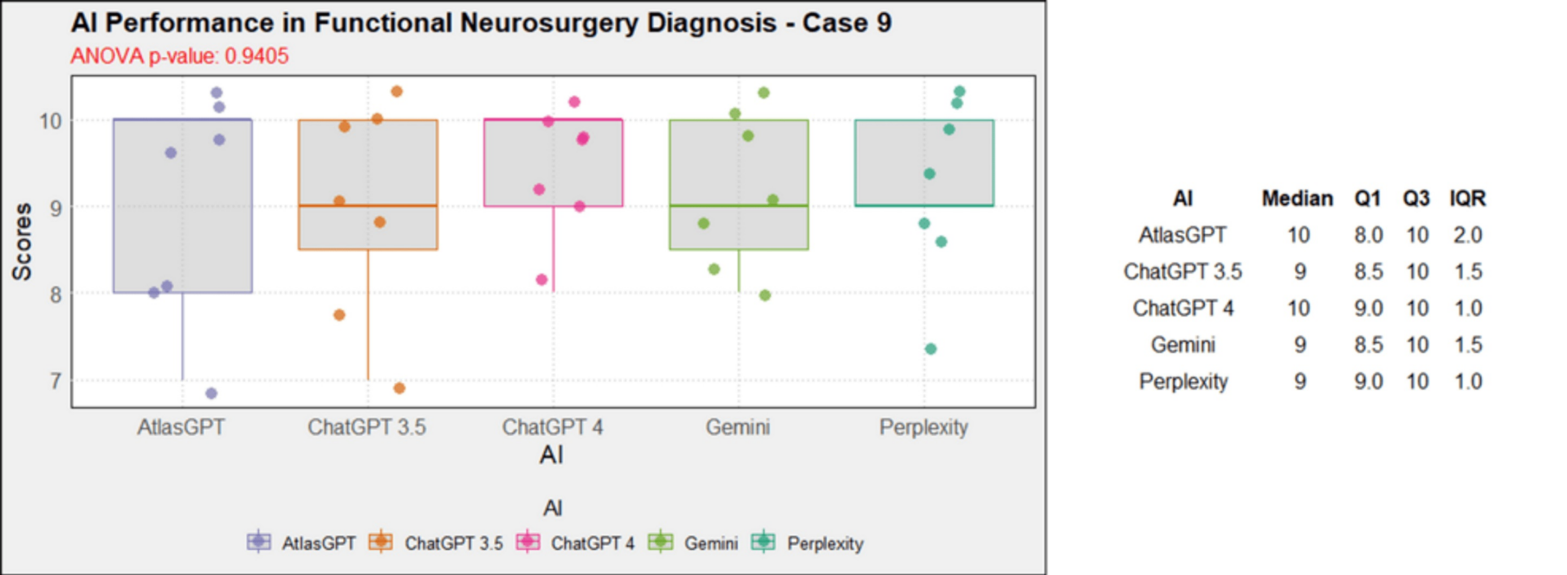
AI models' performance in functional neurosurgery diagnosis - case 9 AI: artificial intelligence; ANOVA: analysis of variance; IQR: interquartile range; Q1: quartile 1; Q3: quartile 3

**Figure 11:**
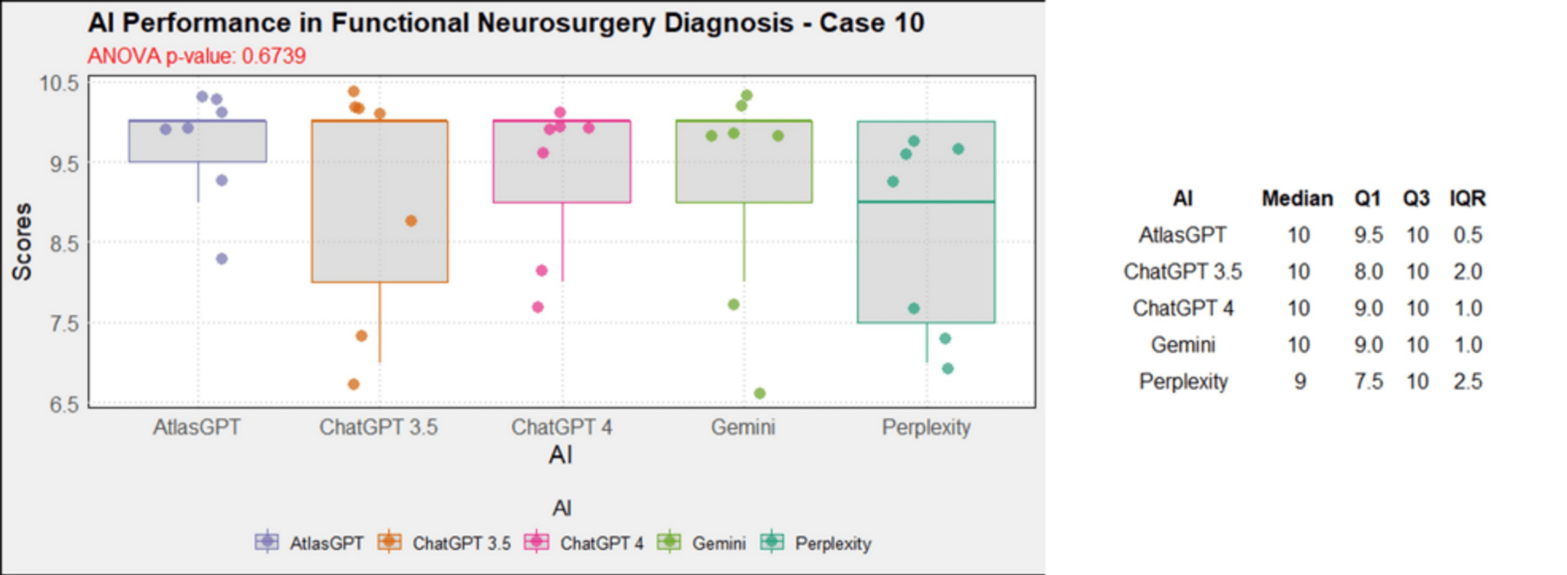
AI models' performance in functional neurosurgery diagnosis - case 10 AI: artificial intelligence; ANOVA: analysis of variance; IQR: interquartile range; Q1: quartile 1; Q3: quartile 3

Comprehensive analysis of the diagnostic and treatment capabilities of AIs revealed varied results: In case 1, there was a significant statistical difference between the AI models (Figure 1). Post-hoc analysis showed AtlasGPT with statistically significant differences compared to Gemini (p=0.0067) and Perplexity (Figure 2), with a median score of 9 (Q1: 9; Q3: 10; IQR: 1). In cases 2 through 10, no statistically significant differences were found between the AI models (p>0.05 for all cases, Figures 3-11).

AI models' treatment recommendations by case

**Figure 12:**
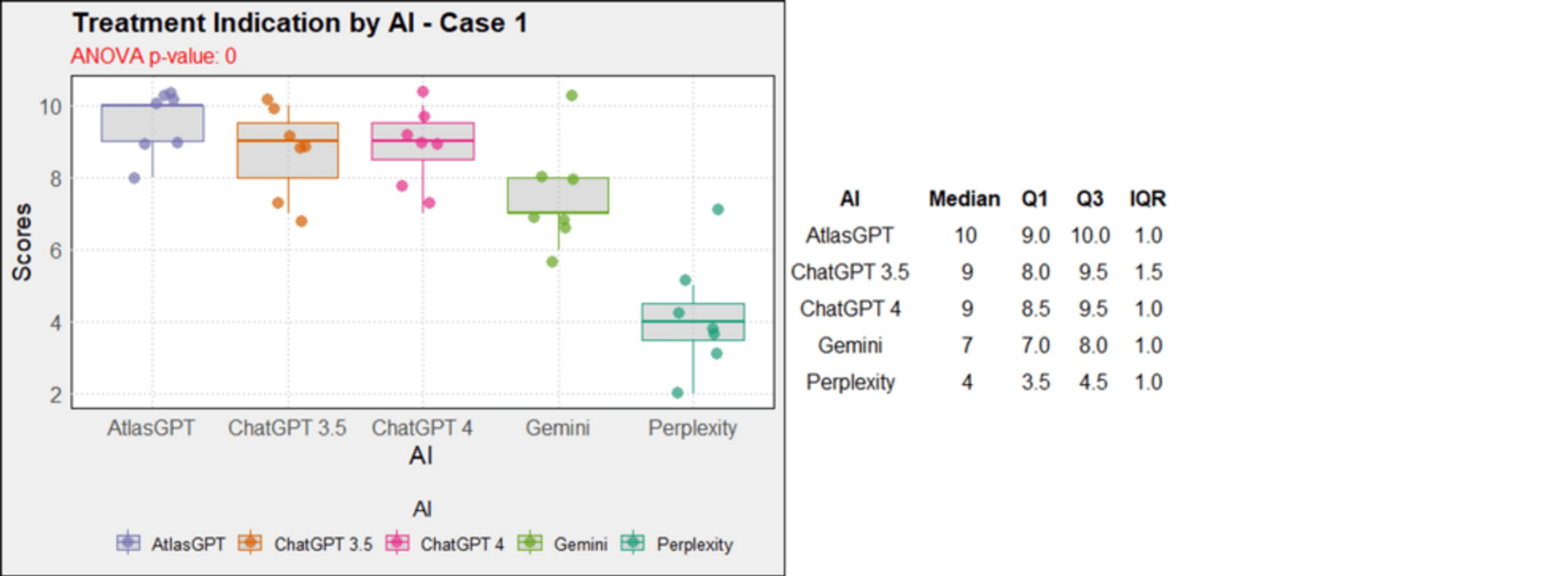
Treatment indication by AI model - case 1 AI: artificial intelligence; ANOVA: analysis of variance; IQR: interquartile range; Q1: quartile 1; Q3: quartile 3

**Figure 13:**
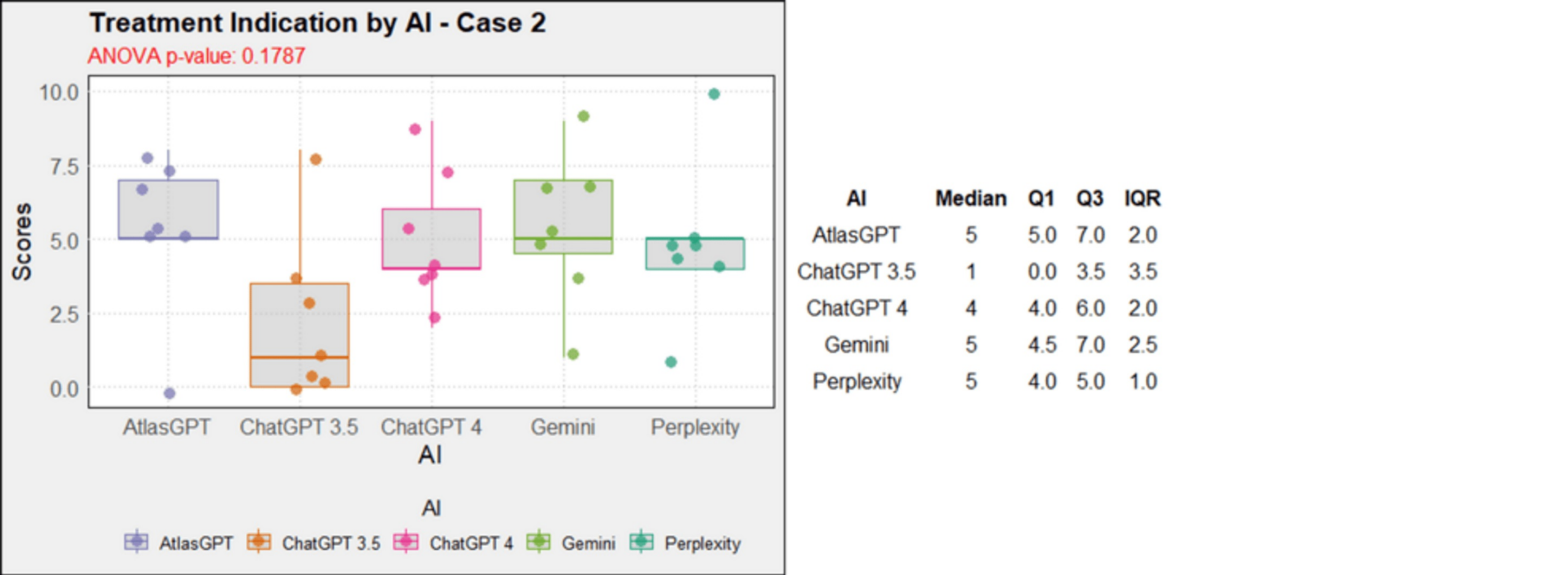
Treatment indication by AI model - case 2 AI: artificial intelligence; ANOVA: analysis of variance; IQR: interquartile range; Q1: quartile 1; Q3: quartile 3

**Figure 14:**
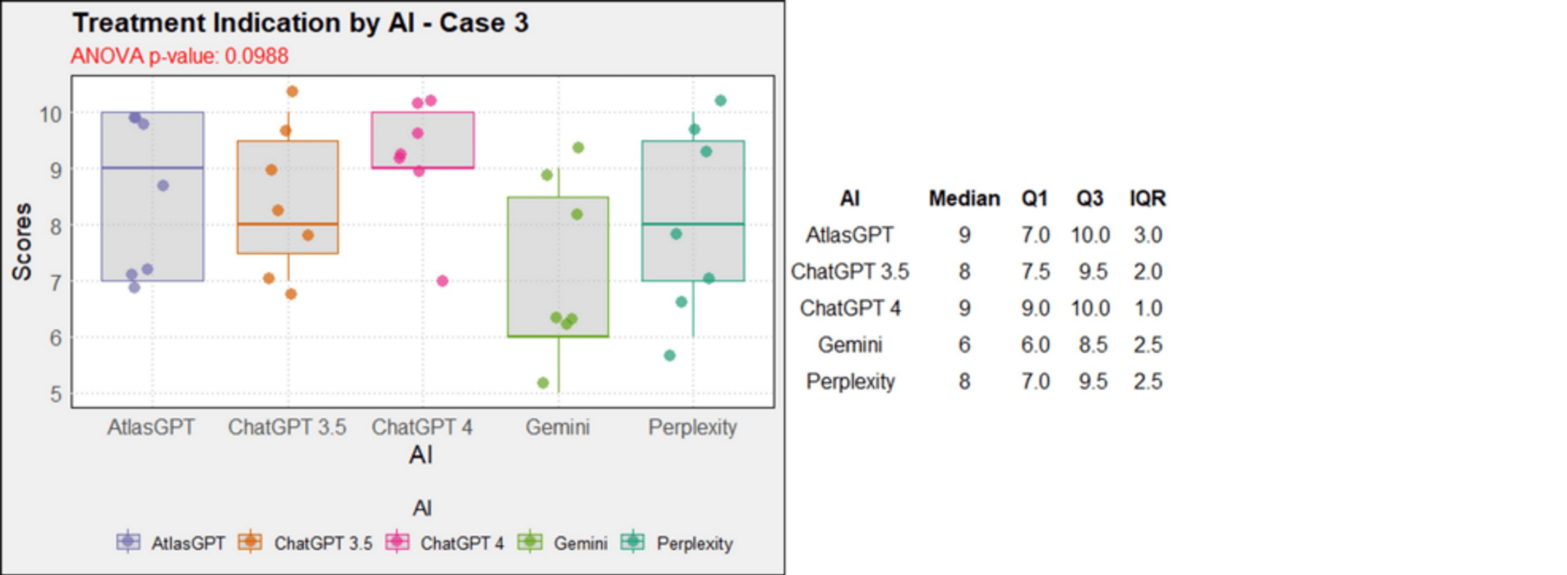
Treatment indication by AI model - case 3 AI: artificial intelligence; ANOVA: analysis of variance; IQR: interquartile range; Q1: quartile 1; Q3: quartile 3

**Figure 15:**
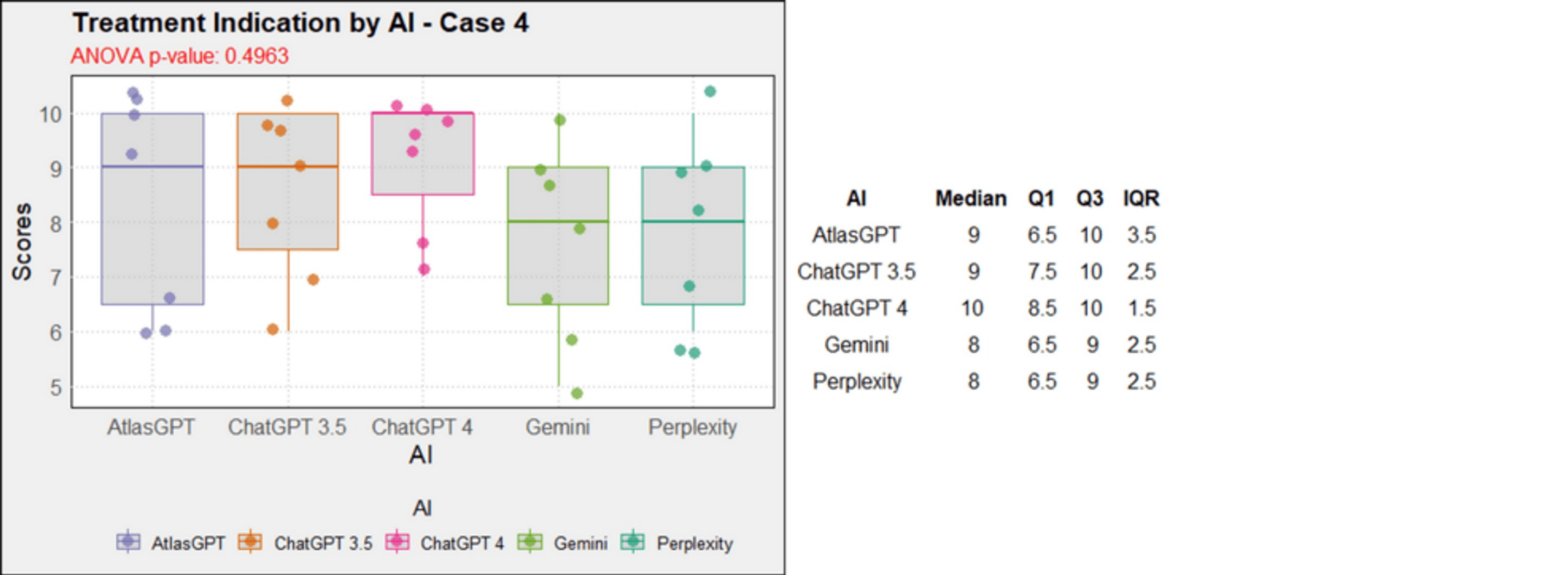
Treatment indication by AI model - case 4 AI: artificial intelligence; ANOVA: analysis of variance; IQR: interquartile range; Q1: quartile 1; Q3: quartile 3

**Figure 16:**
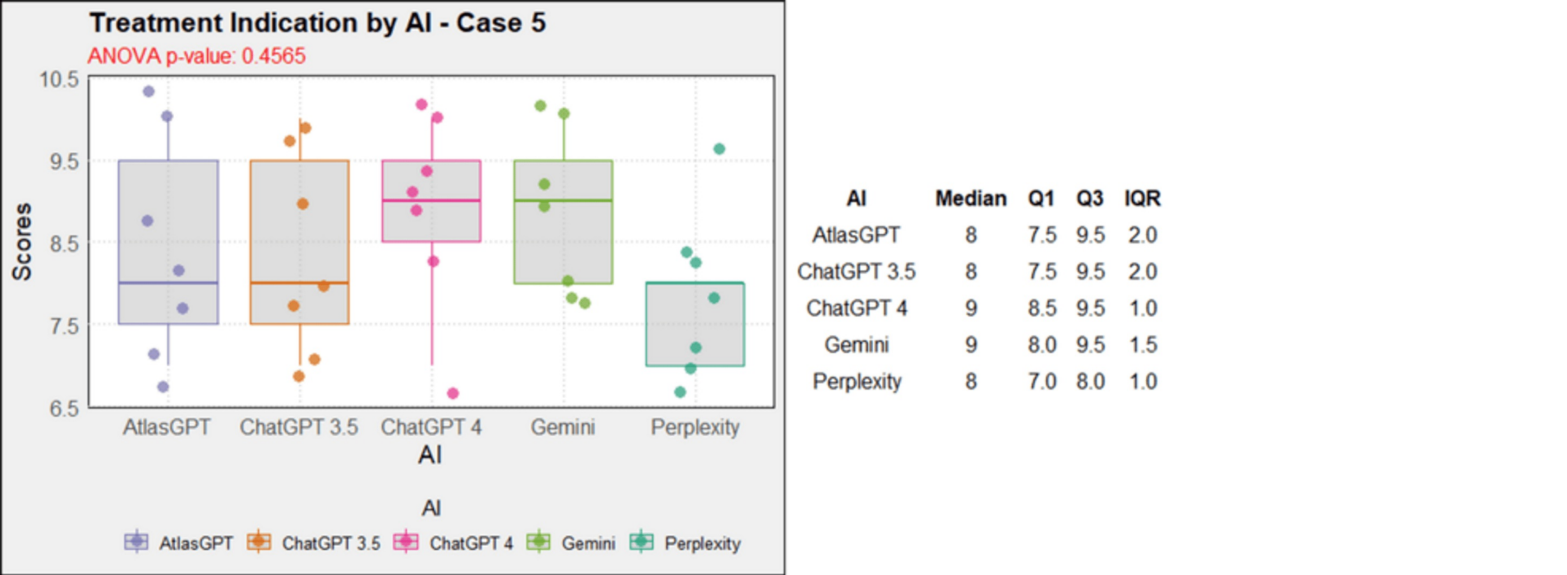
Treatment indication by AI model - case 5 AI: artificial intelligence; ANOVA: analysis of variance; IQR: interquartile range; Q1: quartile 1; Q3: quartile 3

**Figure 17:**
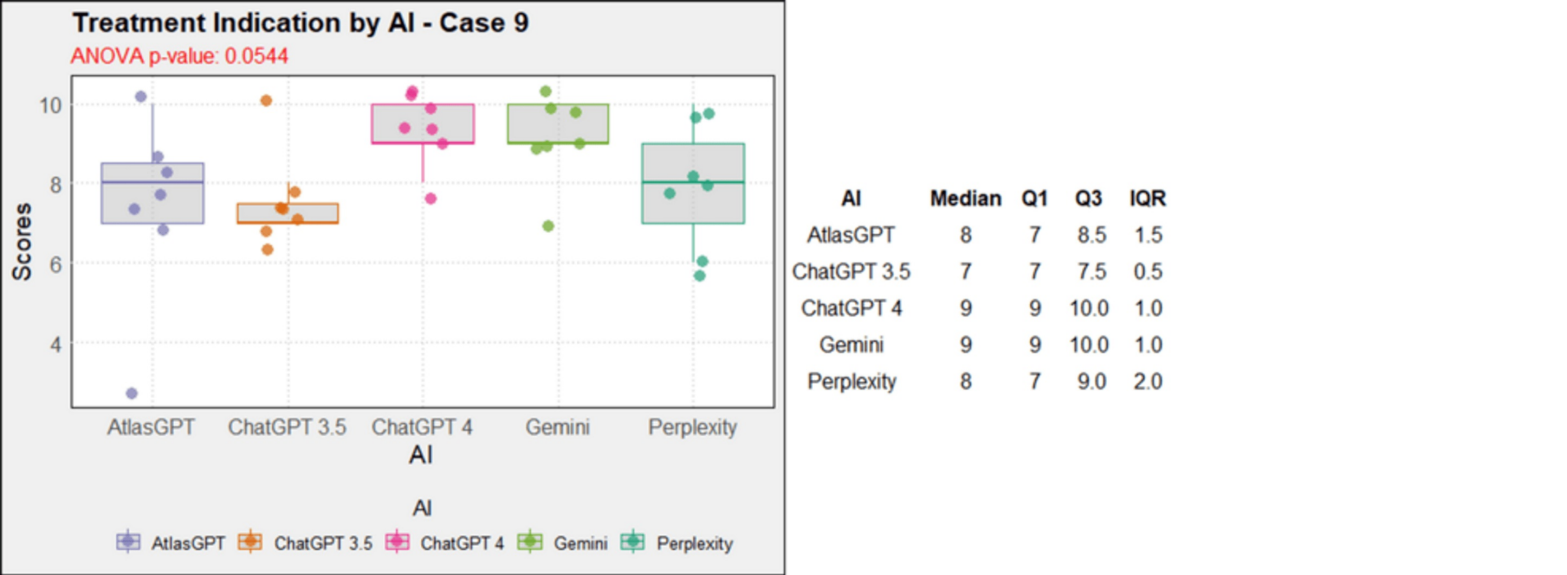
Treatment indication by AI model - case 9 AI: artificial intelligence; ANOVA: analysis of variance; IQR: interquartile range; Q1: quartile 1; Q3: quartile 3

**Figure 18:**
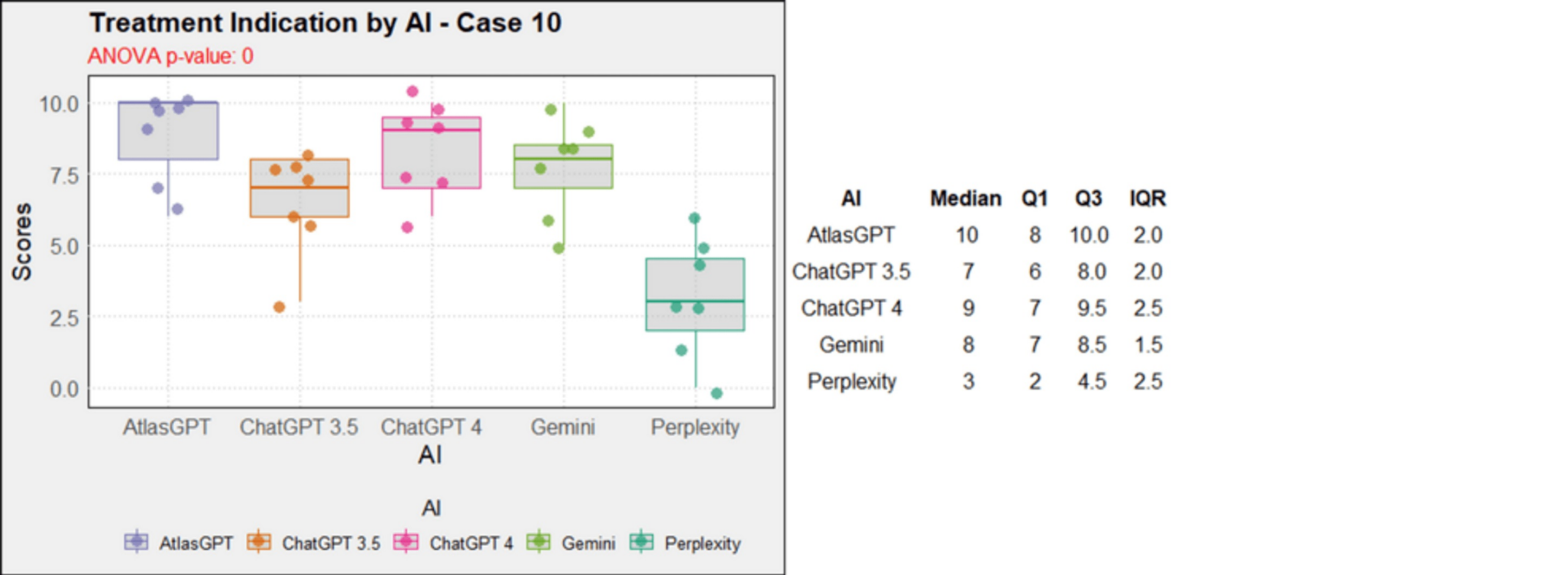
Treatment indication by AI model - case 10 AI: artificial intelligence; ANOVA: analysis of variance; IQR: interquartile range; Q1: quartile 1; Q3: quartile 3

Regarding treatment, in case 1, AtlasGPT, ChatGPT-3.5, ChatGPT-4, and Gemini showed statistically significant differences compared to Perplexity (p=0, Figure 12). In cases 2, 3, 4, 5, 9, and 10, no statistically significant differences were observed between the AIs (p>0.05, Figures 13-18). In case 6, there was a statistical difference between the AIs (p=0.0115); AtlasGPT outperformed Gemini. In case 7, there was also a statistical difference between the AIs (p=0), AtlasGPT and ChatGPT-4 outperformed Perplexity, while ChatGPT-4 also outperformed ChatGPT-3.5. In case 8, ANOVA analysis showed a statistical difference between the AIs (p=0.0025), and Gemini and Perplexity outperformed ChatGPT-3.5. These results highlight significant differences in specific cases, particularly with AtlasGPT demonstrating superior performance compared to other AIs in treatment recommendations.

Global AI comparison

Table 2 presents the results of the ANOVA analysis for diagnosis and treatment recommendations provided by different AI models. The median diagnostic scores for AtlasGPT, ChatGPT-3.5, ChatGPT-4, and Gemini are all 10, with an IQR of 2. Perplexity, though slightly lower with a median of 9, demonstrates a higher IQR of 3, indicating greater variability in its diagnostic capability. For treatment recommendations, AtlasGPT and Gemini have a median treatment score of 8, with IQRs of 3. ChatGPT-3.5 has a median of 7 and an IQR of 2, while ChatGPT-4 and Perplexity have medians of 9, with IQRs of 3. These results highlight the variability and central tendencies of each AI model in providing treatment recommendations, demonstrating distinct patterns in their clinical decision-making processes.

**Table 2:** ANOVA, median, Q1, Q3, and IQR of each AI model regarding the global analysis of diagnosis and treatment indication AI: artificial intelligence; ANOVA: analysis of variance; IQR: interquartile range; Q1: first Quartile; Q3: third quartile

Diagnosis						Treatment					
ANOVA p-value	AI model	Median	Q1	Q3	IQR	ANOVA p-value	AI model	Median	Q1	Q3	IQR
0.03	AtlasGPT	10	8	10	2	<0.01	AtlasGPT	8	7	10	3
	ChatGPT-3.5	10	8	10	2		ChatGPT-3.5	7	6	8	2
	ChatGPT-4	10	8	10	2		ChatGPT-4	9	7	10	3
	Gemini	10	8	10	2		Gemini	8	6	9	3
	Perplexity	9	7	10	3		Perplexity	9	7	10	3

Table 3 provides evidence of a comprehensive post-hoc analysis of AI models' diagnostic and treatment capabilities, revealing varied results: post-hoc combined analysis for diagnosis indicates that AtlasGPT outperformed Perplexity. Post-hoc combined analysis for treatment indicates that AtlasGPT outperformed ChatGPT-3.5, ChatGPT-4.0 outperformed ChatGPT-3.5, and Perplexity outperformed ChatGPT-3.5. These results highlight significant differences in specific cases, particularly with AtlasGPT demonstrating superior performance compared to other AIs in diagnosis and treatment.

**Table 3:** Summary of the post-hoc analysis of diagnosis and treatment global analysis AI: artificial intelligence; diff: difference; lwr: lower bound; upr: upper bound; p adj: adjusted p-value

Diagnosis					Treatment				
AI	diff	lwr	upr	p adj	AI	diff	lwr	upr	p adj
ChatGPT-3.5 - AtlasGPT	-0.21	-1.04	0.61	0.95	ChatGPT-3.5 - AtlasGPT	-1.33	-2.35	-0.31	0.00
ChatGPT-4 - AtlasGPT	-0.09	-0.91	0.74	0.99	ChatGPT-4 - AtlasGPT	0.16	-0.86	1.18	0.99
Gemini - AtlasGPT	-0.13	-0.95	0.70	0.99	Gemini - AtlasGPT	-0.61	-1.63	0.40	0.46
Perplexity - AtlasGPT	-0.86	-1.68	-0.03	0.04	Perplexity - AtlasGPT	0.09	-0.93	1.10	0.99
ChatGPT-4 - ChatGPT-3.5	0.13	-0.70	0.95	0.99	ChatGPT-4 - ChatGPT-3.5	1.49	0.47	2.50	< 0.01
Gemini - ChatGPT-3.5	0.09	-0.74	0.91	1.00	Gemini - ChatGPT-3.5	0.71	-0.30	1.73	0.30
Perplexity - ChatGPT-3.5	-0.64	-1.47	0.18	0.21	Perplexity - ChatGPT-3.5	1.41	0.40	2.43	< 0.01
Gemini - ChatGPT-4	-0.04	-0.87	0.78	1.00	Gemini - ChatGPT-4	-0.77	-1.79	0.25	0.23
Perplexity - ChatGPT-4	-0.77	-1.60	0.05	0.08	Perplexity - ChatGPT-4	-0.07	-1.09	0.95	0.99
Perplexity - Gemini	-0.73	-1.55	0.10	0.11	Perplexity - Gemini	0.70	-0.32	1.72	0.32

**Figure 19:**
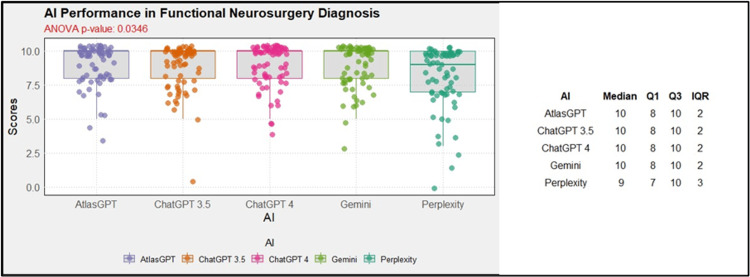
Global AI performance in diagnosis AI: artificial intelligence; ANOVA: analysis of variance; IQR: interquartile range; Q1: first Quartile; Q3: third quartile

**Figure 20:**
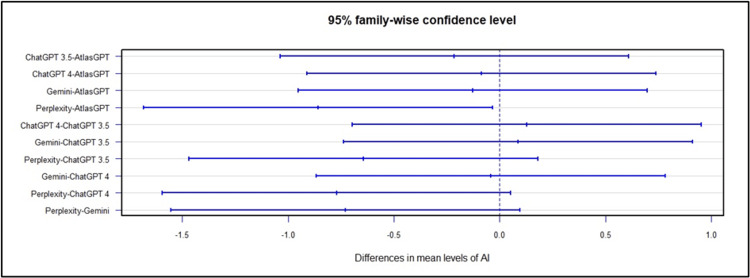
Global post-hoc analysis for AI performance in diagnosis AI: artificial intelligence

Considering the capacity to diagnose of the AIs, ANOVA global analysis showed a statistical difference between the AIs (p=0.03, Figure 19). Further post-hoc analysis showed a better result for AtlasGPT, outperforming Perplexity (MD = -0.86; 95% CI: -1.68 to -0.03; p=0.04; Figure 20).

**Figure 21:**
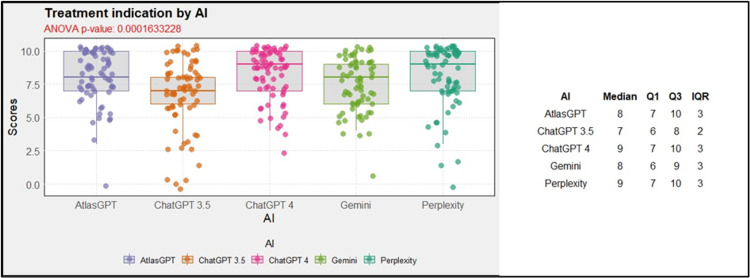
AI global performance in treatment indication AI: artificial intelligence; ANOVA: analysis of variance; IQR: interquartile range; Q1: first Quartile; Q3: third quartile

**Figure 22:**
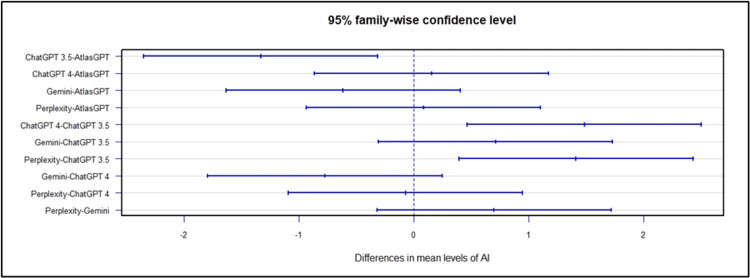
Global post-hoc analysis for AI performance in treatment indication AI: artificial intelligence

Concerning the treatment indications of the AIs, ANOVA global analysis showed a statistical difference between the AIs (p<0.01, Figure 21). Post-hoc combined analysis for treatment indicates that Atlas GPT outperformed ChatGPT-3.5, (MD = -1.33; 95% CI: -2.35 to -0.31; p<0.01; Figure 22), ChatGPT-4.0 outperformed ChatGPT-3.5 MD = 1.49; 95% CI: 0.47 to 2.50; p<0.01; Figure 22), and Perplexity outperformed Chat GPT 3.5 (MD = 1.41; 95% CI: 0.40 to 2.43; p<0.01; Figure 22).

## Discussion

The results of this study highlight the significant potential of AI models in functional neurosurgery, demonstrating their ability to assist in diagnosis and therapeutic planning. The AI models AtlasGPT, ChatGPT-3.5, ChatGPT-4.0, Gemini, and Perplexity were evaluated to determine their effectiveness in a series of complex clinical cases. Overall, the AI models demonstrated high diagnostic accuracy, with median scores close to or equal to 10. However, there was considerable variation in treatment recommendations among the different models. Notably, AtlasGPT stood out for its superior performance in both diagnosis and therapeutic recommendations, especially compared to ChatGPT-3.5 and Perplexity. This disparity suggests that different AI models may have distinct strengths, potentially due to variations in training data and algorithms used, highlighting the need for ongoing critical evaluation of these technologies before their widespread clinical implementation [5,6].

For instance, in advanced Parkinson's cases, all models correctly identified the condition and recommended DBS. However, in refractory epilepsy cases, AtlasGPT and ChatGPT-4.0 went beyond standard recommendations, suggesting alternatives such as RNS and LITT, while Perplexity remained more conservative. This indicates that certain AI models have the capability to not only replicate existing diagnoses but also expand therapeutic options with more personalized and potentially more effective approaches [6,7,8].

Statistical analysis, including ANOVA and post-hoc tests, revealed statistically significant differences in the models' performance. AtlasGPT, in particular, frequently outperformed other models in terms of diagnostic accuracy and the quality of treatment recommendations. The variability in performance among the models suggests that while some AI models are more consistent and reliable in their recommendations, others may require further refinement. Advances in learning algorithms and the diversity of training data are critical factors for the success of AIs in clinical practice [7].

In addition to technical considerations, the use of AI in medicine raises important ethical issues. Patient data privacy is a central concern, especially given the need for large volumes of data to train AI models. Algorithm transparency is essential to ensure the trust of healthcare professionals and patients, allowing decisions to be auditable and verifiable. In many cases, machine learning models have outperformed clinical experts in predictive accuracy, demonstrating the potential of AIs to overcome human limitations in certain tasks. However, the possibility of "artificial hallucinations" - situations where AI models generate incorrect or fabricated information - underscores the need for continuous human oversight to prevent significant errors [8].

Responsibility for medical errors caused by automated decisions is also a complex ethical issue. Establishing clear guidelines on who is responsible for these decisions is crucial to ensure that AI-based recommendations are used safely and ethically. The literature suggests that combining human expertise with AI models may be the most effective approach, maximizing benefits and minimizing the risks associated with relying solely on one of these approaches [9].

The integration of AI into clinical practice can offer substantial benefits, such as reducing medical errors and personalizing treatment, which can be adjusted based on real-time data and updated evidence. However, the development of these technologies must be accompanied by rigorous and continuous evaluation, addressing both technical challenges and ethical issues. Interdisciplinary collaboration and the establishment of a robust ethical and legal framework are fundamental for the safe and effective implementation of these technologies in medical practice [8].

To sum up, this study underscores the transformative potential of AI technologies in functional neurosurgery. Nevertheless, incorporating these advancements into clinical practice requires an ongoing and thorough evaluation, addressing both technical obstacles and ethical concerns. Interdisciplinary collaboration will be essential to ensure that AI technologies are implemented safely, effectively, and equitably, ultimately delivering tangible benefits to patients.

Limitations

This study has several limitations that should be considered. The selection of clinical cases was based on examples available in the literature, which may not represent the full complexity encountered in daily clinical practice. Additionally, the evaluations were conducted by a panel of experts, introducing a level of subjectivity to the analysis of AI responses. Finally, the lack of a real clinical environment may limit the generalizability of the results.

## Conclusions

The results of this study highlight the transformative potential of AI technologies in functional neurosurgery, with ChatGPT-4.0 and AtlasGPT emerging as powerful tools to help physicians in daily practice. However, the integration of these innovations into clinical practice must be accompanied by rigorous and continuous evaluation, addressing both technical challenges and ethical issues. This ongoing critical evaluation is necessary to ensure that these models are implemented safely and effectively, maximizing their potential benefits while minimizing associated risks. Interdisciplinary collaboration will be crucial to ensure that AI technologies are applied safely, effectively, and equitably, providing real benefits to patients.
